# Network-based machine learning and graph theory algorithms for precision oncology

**DOI:** 10.1038/s41698-017-0029-7

**Published:** 2017-08-08

**Authors:** Wei Zhang, Jeremy Chien, Jeongsik Yong, Rui Kuang

**Affiliations:** 1 Department of Computer Science and Engineering, University of Minnesota Twin Cities, Minneapolis, MN USA; 2 Department of Cancer Biology, University of Kansas Medical Center, Kansas City, KS USA; 3 Department of Biochemistry, Molecular Biology and Biophysics, University of Minnesota Twin Cities, Minneapolis, MN USA

## Abstract

Network-based analytics plays an increasingly important role in precision oncology. Growing evidence in recent studies suggests that cancer can be better understood through mutated or dysregulated pathways or networks rather than individual mutations and that the efficacy of repositioned drugs can be inferred from disease modules in molecular networks. This article reviews network-based machine learning and graph theory algorithms for integrative analysis of personal genomic data and biomedical knowledge bases to identify tumor-specific molecular mechanisms, candidate targets and repositioned drugs for personalized treatment. The review focuses on the algorithmic design and mathematical formulation of these methods to facilitate applications and implementations of network-based analysis in the practice of precision oncology. We review the methods applied in three scenarios to integrate genomic data and network models in different analysis pipelines, and we examine three categories of network-based approaches for repositioning drugs in drug–disease–gene networks. In addition, we perform a comprehensive subnetwork/pathway analysis of mutations in 31 cancer genome projects in the Cancer Genome Atlas and present a detailed case study on ovarian cancer. Finally, we discuss interesting observations, potential pitfalls and future directions in network-based precision oncology.

## Introduction

**Fig. 1:**
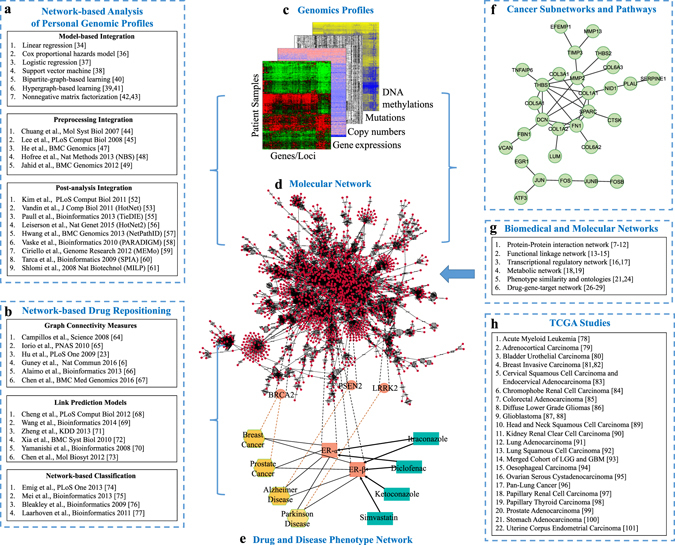
Overview of the methods for network-based precision oncology. **a** The methods for integration of patient genomic data and molecular networks grouped under the three scenarios of data analysis pipelines. **b** The methods for integration of drug–drug similarities, drug–target relations and target–target relations for drug repositioning, grouped under three algorithmic categories. **c** Patient genomic profiles describe the genomic landscape of each patient sample. **d** The patient genomic profiles are integrated with a molecular network, the human protein–protein interaction (PPI) network in the example. **e** Drug and disease phenotypes are modeled in a network with connections to the target genes in the PPI network. **f** An example of cancer subnetworks associated with recurrent ovarian cancer.^36^ **g** Resources of biomedical and molecular networks. **h** List of the TCGA cancer studies

The revolutionary large-scale genomic and sequencing technologies developed in the past two decades have enabled an understanding of cancer biology in individual tumors for personalized treatment. Coordinated national and international efforts for cancer genome projects have been launched to characterize tens of thousands of individual tumors by somatic mutation, gene expression, copy number variation, DNA methylation, and various other types of genomic and epigenomic aberrations.^1, 2^ The large volume of accumulated cancer genomic data has facilitated the identification of precise oncogenes and tumor suppressors for the development of personalized therapeutic strategies. One of the well-recognized new observations in these studies is that cancer is better characterized by frequently mutated or dysregulated pathways than driver mutations, which are often distinct in the tumors of the same type.^3^ For example, studies have reported that only a few altered genes occur in more than 10% of the samples and that many other altered genes occur in less than 5% of the samples in the same tumor type.^4^ Furthermore, certain cancer types, such as prostate cancer and pediatric cancers, are not driven by a few somatic mutations or copy number variations, and the mechanism might be better understood in the context of systems biology.^4^ This important observation has led to a great effort to develop a collection of network-based computational methods to detect cancer pathways or subnetworks by integration of various genomic data, as shown in Fig. 1a, and these methods can be classified into three categories depending on the scenario of applying the analysis pipeline.

Network-based analysis has also attracted considerable attention in drug repositioning to reduce the cost of new drug development by using repositioned existing drugs on novel targets in drug–target networks for precision oncology.^5^ Based on the hypothesis that drugs tend to be more effective on target genes within or in the vicinity of a disease module in a molecular network,^5, 6^ several network-based approaches have been used to explore networks of drugs, diseases and targets to reposition drugs for new targets, as listed in Fig. 1b. In these methods, the drug–target relations can be inferred by various measures in the network, combining drug–drug, drug–target, drug–disease and disease–gene relations as shown in the drug–disease–target network in Fig. 1d, e. As summarized in Fig. 1b, these methods can be classified into three categories based on the underlying computational formulation: methods using graph connectivity measures, link prediction methods and network-based classification methods.

The focus of this review article is to provide a comprehensive and unified survey of machine learning and graph theory algorithms for network analysis in precision oncology. We compare the methods by their distinctions in the methodology and mathematical formulations such that the methods can be better applied and improved appropriately for precision oncology. An overview of this article is given in Fig. 1. We not only review the resources of biomedical and molecular networks listed in Fig. 1g and the network-based methods listed in Fig. 1a, b but also present a comprehensive network-based pathway analysis of mutations in 31 cancer genome projects in the Cancer Genome Atlas (TCGA) list in Fig. 1h and a case study on ovarian cancer to show the promise of applying network-based analysis.

## Biomedical and molecular networks

In the literature, various biological and biomedical network databases have been compiled to support network analysis. Typically, the databases have been curated by the integration of high-throughput experimental screening results from studies in the literature and possibly computational predictions supervised by expert knowledge. The networks represent the collections of molecules, phenotypes and drugs as nodes and their relations as edges in graphs. In Table 1, we enumerate existing molecular networks, phenotype similarity networks or ontologies, and drug–target networks and the resources for obtaining these networks. The properties of these networks, including their nodes, edges and graph structures, are also shown in Table 1.

1. Molecular networks: Biological molecular networks describe relations among molecules, such as protein–protein interactions, gene co-expression, functional similarities, regulatory relations or biochemical reactions. The new-generation high-throughput technologies have provided extensive content to construct such molecular networks. Protein–protein interaction networks are available from several well-maintained databases.^7–12^ Primarily, these networks include physical interactions determined by experiments and computationally derived interactions. Proteome-wide protein–protein interactions capture the interplay among proteins based on the functional associates from co-membership of protein complexes and pathways. A functional linkage network is a more comprehensive compilation of functional relations, physical interactions and co-expression in one network.^13–15^ A transcriptional regulatory network models the molecular interactions between transcript factors/microRNA and target genes to regulate transcript expression.^16, 17^ A transcriptional regulatory network is a directed graph in which the edges connect a regulator to its targets. A cellular metabolic network can be constructed by the co-membership of biochemical reactions among metabolites and enzymes.^18, 19^ Several graph structures can be used to represent metabolic pathways, e.g., labeled directed graphs, unions of bipartite graphs (per reaction) and hypergraphs, depending on the level of detail of metabolic reactions to be modeled with the graph.^20^
2. Phenotype similarity networks and ontologies: Phenotypes, particularly disease phenotypes, are of special interest for cancer studies. The analysis of diseases in the context of other related diseases can offer insight into their genotypic drivers. Online Mendelian Inheritance in Man (OMIM) is a comprehensive compendium of human genes, genetic phenotypes and documentation of their phenotype–gene associations.^21^ Phenotype similarity networks can be constructed based on the genetic resemblance^22^ or the synopsis of the diseases and sometimes by mRNA expression.^23^ Human Phenotype Ontology (HPO) is another more comprehensive organization of all human disease phenotypes in an ontology.^24^ The ontology is a directed acyclic graph that can be used as a network structure for learning phenotype–gene associations.^25^
3. Drug–target and drug–drug networks: Drug–target associations can be modeled by a bipartite network with connections between the drugs and their targets. The drug–target pairs are typically derived from FDA-approved or experimental drugs and their human protein targets available from various drug databases.^26–29^ Several different types of drug–drug similarity networks have been derived for drug repositioning. Drug–drug relations can be inferred based on similarity of molecular basis, chemical substructure, and phenotypes, such as known drug-indication relations, co-membership in drug combinations, and co-morbidity of diseases.^30^

**Table 1:** List of molecular and biomedical networks

**Protein-Protein Interaction Network**	**Nodes: proteins; Edges: physical contacts or predicted protein–protein bindings; Typical Structure: undirected unweighted graph**
HPRD(7)	“Human Protein Reference Database provides curated human-specific protein interactions; currently >40,000 interactions for >30,000 protein entries. HPRD is used as a browser for interactions, protein annotations, motifs and domains.”
BioGRID (8)	BioGRID is a curated database of interactions, derived from the literature. It contains 1,412,140 protein and genetic interactions, 27,745 chemical associations and 38,559 post translational modification from major organism species.
MINT (9)	“A searchable molecular interaction database with total of 125,000 interactions reported in peer-reviewed journals.” Most of the interactions are from yeast, human and mouse.
DIP (10)	“The database of interacting proteins (DIP) is a database with catalogs experimentally determined protein–protein interactions.” It contains 81,731 interactions for 28,868 proteins from 834 organisms.
STRING (11)	A database of known and predicted protein–protein interactions. “The interactions include direct (physical) and indirect (functional) associations; they stem from computational prediction, from knowledge transfer between organisms, and from interactions aggregated from other databases.”
IntAct (12)	“A molecular interaction database populated by data either curated from the literature or from direct data depositions. It contains approximately 658,000 curated binary interaction evidences from overall 14,451 publications.”
**Functional Linkage Network**	**Nodes: genes; Edges: functional relations; Typical Structure: undirected weighted graph**
HEFalMp (15)	“A human gene functional network was constructed by a regularized Bayesian integration system. The network contains maps of functional activity and interaction networks in over 200 areas of human cellular biology with information from 30,000 genome scale experiments.”
Co-expression Network (13, 14)	A gene co-expression network is constructed by looking for pairs of genes which show a similar expression pattern across samples by some co-expression measure.
**Transcriptional Regulatory Network**	**Nodes: genes;** **Edges** **:** **regulatory relations;** **Typical Structure** **: directed graph**
TRRUST (16)	“A manually curated database of human transcriptional regulatory network. It contains 8015 transcriptional regulatory relationships between 748 human transcription factors (TFs) and 1975 non-TF genes, derived from 6175 PubMed articles.”
RegNetwork (17)	A database of transcriptional and post-transcriptional human and mouse regulatory networks. It collects knowledge-based regulatory relationships and certain potentially regulatory relationships between the two regulators and targets.
**Metabolic Network**	**Nodes** **: metabolites and enzyme proteins;** **Edges** **: biochemical reaction and regulation and metabolic pathways;** **Typical Structures:** **labeled directed graph, union of bipartite graphs (per reaction), directed/undirected hypergraphs**
HMDB (18)	“A database contains information about small molecule metabolites found in the human body. It contains experimental MS/MS data for over 5700 compounds, experimental NMR data for over 1300 compounds and GC/MS spectral and retention index data for more than 780 compounds.”
MetaCyc (19)	“A curated database of experimentally elucidated metabolic pathways from all domains of life. It contains 2491 pathways involved in both primary and secondary metabolism, as well as associated metabolites, reactions, enzymes, and genes from 2816 different organisms.”
**Phenotype Network and Ontologies**	**Nodes** **: diseases and their phenotypes;** **Edges** **: causal relation and subclass-of;** **Structure** **: directed acyclic graph**
OMIM (21)	“OMIM is a database of human genes and genetic disorders and traits, with a particular focus on the gene-phenotype relationship.” It contains approximately 8000 phenotypes and 15,000 genes.
HPO (24)	“HPO serves as a standardized vocabulary of phenotypic abnormalities that have been seen in human disease.” It currently focuses on monogenic diseases listed in OMIM, Orphanet, DECIPHER and other medical literature.
**Drug-Target Network**	**Nodes:** **drugs and target proteins/DNAs/other biological targets;** **Edges:** **physical binding;** **Structure:** **bi-partite graph**
DragBank (26)	“A bioinformatics and cheminformatics resource that combines detailed drug data with comprehensive drug target information. It contains 8250 drug entries including 2016 FDA-approved small molecule drugs, 229 FDA-approved biotech drugs, 94 nutraceuticals and over 6000 experimental drugs.”
ChEMBL (27)	“ChEMBL is a bioactivity database containing information manually extracted from the medicinal chemistry literature.” It contains the information extracted from >51,000 publications, with >9000 targets of which 2827 are human protein targets.
TTD(28)	“A database contains the known and explored therapeutic protein and nucleic acid targets, the targeted disease, pathway information and the corresponding drugs directed at each of these targets.” The database currently contains 2025 targets, 17,816 drugs, and 3681 multitarget agents.
KEGG DRUG (29)	“A comprehensive drug information resource for approved drugs in Japan, USA, and Europe unified based on the chemical structures and/or the chemical components, and associated with target, metabolizing enzyme, and other molecular interaction network information.”

## Network-based analysis of personal genomic profiles

**Fig. 2:**
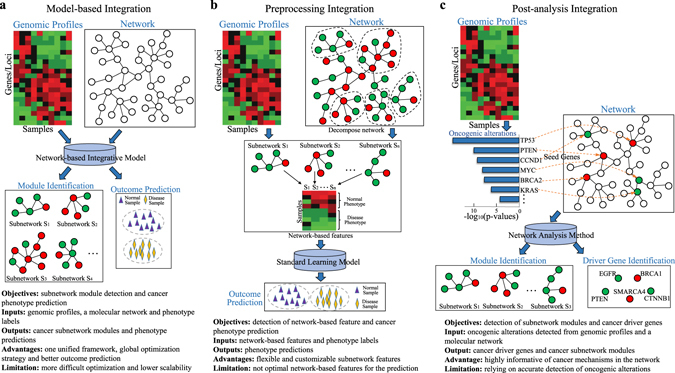
Three scenarios for the integration of genomic data with molecular networks. **a** Model-based integration formulates one unified learning framework regularized by a graph Laplacian. The output of the model is network modules enriched by the selected genomic features and a prediction of treatment outcome/cancer phenotype. **b** Preprocessing integration consists of the following two steps: The first step detects subnetworks that differentiate the contrasted patient groups by the genomic features; in the second step, the subnetwork features are then fed into a standard learning model to generate predictions. **c** Post-analysis integration of oncogenic alterations in the network also consists of two steps. The oncogenic alterations are first detected across the patient profiles, and then the altered genes/loci are mapped to the network as seed genes for the module analysis. For each scenario, the objectives of the approach, the inputs and outputs of the network-based analysis models/methods, and the advantages/limitations of each approach are also provided

The goal of applying network-based analysis to personal genomic profiles is to identify aberrant network modules that are both informative of cancer mechanisms and predictive of cancer phenotypes. These methods can be classified into three categories based on the design of the analysis pipeline in different scenarios, as shown in Fig. 2. In these scenarios, the detection of the network modules facilitates two other goals: predicting cancer phenotypes and detecting driver genes. Depending on how the network information is processed in the pipeline, the inputs and the outputs to the predictive models or network analysis methods can differ. Below, we describe the three categories of the methods listed in Fig. 1a and then discuss the advantages and limitations of each of the categories.

### Model-based integration of whole-genomic profiles and a network

**Fig. 3:**
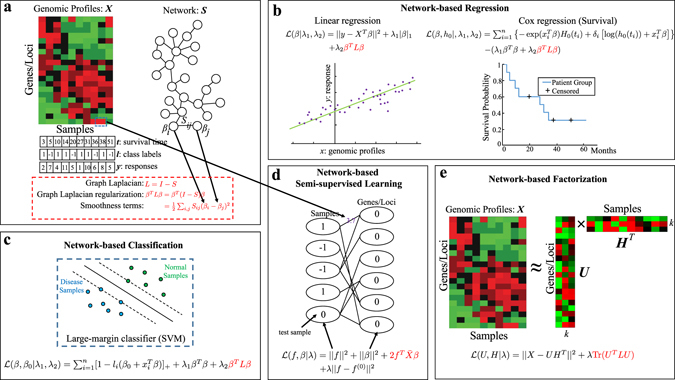
Model-based integration of whole-genomic profiles and a molecular network. **a** The patient genomic profiles ***X*** along with the clinical information: the survival time, two patient subgroups for classification and treatment response of each individual patient are shown. The network ***S*** is typically integrated into the genomic profile analysis with a graph Laplacian regularization. The formulas of the graph Laplacian and its regularization are shown below. The graph Laplacian regularization can be rewritten as summation of pairwise smoothness terms that promote smoothness among the connected genomic features in the network. **b** The network-based linear regression and Cox regression models are illustrated in the figure with the graph Laplacian regularization term added to the original cost functions. **c** Network-based classification is illustrated by a network-based SVM to classify the samples. **d** Network-based semi-supervised learning models classify samples and detect disease markers on a bipartite graph. The edges between samples and genomic features are weighted by the genomic profiles, and semi-supervised learning is based on the bipartite graph Laplacian. **e** Network-based factorization models factorize the genomic profile ***X*** into the product of two matrices, ***U*** and ***H***, which cluster patient samples and learn the latent features in the genomic profiles

Model-based integration formulates a single unified machine learning framework to integrate genomic profiles with a network as illustrated in Fig. 2a. The core technique is to introduce a network-based regularization into machine learning models such that the coefficients learned on the feature variables form dense subnetworks. The most commonly used network-based regularization is the graph Laplacian regularizer shown in Fig. 3a. The graph Laplacian was first introduced for spectral graph analysis^31^ and then used for semi-supervised learning in machine learning.^32, 33^ The graph Laplacian regularization is a summation of smoothness terms on the variables to encourage similar coefficients on the genes or other genomic features that are connected in the network. Below, we describe the graph Laplacian regularized methods in different learning frameworks as shown in Fig. 3b–e. To precisely describe the models, we also list all the necessary notations in Table 2 and the exact mathematical formulations of the methods in Supplementary Table S2.

**Table 2:** Notations

Notation	Definition
*n*, *m*	# of samples and features (e.g., genes), respectively.
***X*** ∈ ${{\Bbb R}^{m \times n}}$	genomic profile matrix.
***β*** ∈ ${{\Bbb R}^{m \times 1}}$	coefficients of features to be learned by the model.
${\boldsymbol{y}} \in {{\Bbb R}^{n \times 1}}$	responses for regression or labels for classification, ***y*** = (*y*_1_, …, *y*_*n*_)^T^.
${\boldsymbol{W}} \in {{\Bbb R}^{m \times m}}$	symmetric adjacency matrix of an undirected molecular network.
***D***_***x***_	diagonal matrix with vector ***x*** on the diagonal.
${\boldsymbol{S}} \in {{\Bbb R}^{m \times m}}$	normalized symmetric adjacency matrix: ${\boldsymbol{S}} = {\boldsymbol{D}}_{\boldsymbol{w}}^{ - \frac{1}{2}}{\boldsymbol{WD}}_{\boldsymbol{w}}^{ - \frac{1}{2}}$, where **w** is the row sum of ***W***.
${\boldsymbol{L}} \in {{\Bbb R}^{m \times m}}$	normalized graph Laplacian: ***L*** = ***I*** − ***S***.
***β*** ^T^ ***Lβ***	graph Laplacian regularization: ${{\boldsymbol{\beta }}^{\rm T}}\ {\boldsymbol{L\beta }} = \frac{1}{2}\mathop {\sum}\nolimits_{i,j} {{S_{i,j}}{{\left( {{\beta _i} - {\beta _j}} \right)}^2}}$.
${{\boldsymbol{f}}^{\left( {\bf{0}} \right)}} \in {{\Bbb R}^{n \times 1}}$	initialization for semi-supervised learning: ${{\boldsymbol{f}}^{\left( {\bf{0}} \right)}} = {\left\{ {f_1^{\left( 0 \right)}, \ldots,f_i^{\left( 0 \right)}, \ldots,f_n^{\left( 0 \right)}} \right\}^{\rm{T}}}$, where ${\boldsymbol{f}}_i^{\left( {\bf{0}} \right)} \in \left\{ { - 1,0, + 1} \right\}$. 0 is assigned if there are additional unlabeled data for semi-supervised learning.
${\boldsymbol{f}} \in {{\Bbb R}^{n \times 1}}$	Predictions by semi-supervised learning: ${\boldsymbol{f}} = {\left\{ {{f_1}, \ldots,{f_n}} \right\}^{\rm{T}}}$.
*λ*, *λ*_1_, *λ*_2_	positive hyper-parameters to weight the cost terms.

In Fig. 3b, the widely used regression and survival models are extended to include the graph Laplacian constraint for the analysis of genomic data. The paper^34^ proposed a network-constrained linear regression procedure that combines a graph Laplacian constraint with the *L*_1_-norm sparse linear regression to capture the relations among the regression coefficients.^35^ This network-based linear regression is equivalent to a standard LASSO optimization problem.^34^ The paper^36^ proposed a network-based Cox proportional hazards model (Net-Cox) for survival analysis. In Cox regression, the objective is to learn the regression coefficients *β* and the baseline hazard function *h*_0_(*t*) such that the instantaneous risk of an event at time *t* for a patient ***x***_*i*_ can be estimated by $h\left( {t|{{\boldsymbol{x}}_i}} \right) = {h_0}(t)exp\left( {{\boldsymbol{x}}_i^{\rm{T}}\beta } \right)$. Similarly, the graph Laplacian constraint is introduced on the regression coefficients *β*. By alternating between maximization with respect to *β* and *h*_0_(*t*), a local optimum can be found.

As shown in Fig. 3c, the graph Laplacian constraint can also be introduced into linear classification models such as logistic regression^37^ and support vector machines (SVMs).^38^ Given the binary response vector ***y*** = (*y*_1_, ..., *y*_*n*_)^T^ with *y*_*i*_ ∈ {1, 0}, a Bernoulli likelihood function minus both the *L*_1_-norm and the graph Laplacian constraints is maximized to learn the linear coefficients. In the model, $p\left( {{{\boldsymbol{x}}_i}} \right) = \frac{{{\rm{exp}}\left( {{\beta _0} + {\boldsymbol{x}}_i^{\rm{T}}\beta } \right)}}{{1 + {\rm{exp}}\left( {{\beta _0} + {\boldsymbol{x}}_i^{\rm{T}}\beta } \right)}}$ is the probability that the *i*th sample is in class 1. The elastic-net procedure can be applied to maximize the regularized cost function. The paper^38^ proposed a network-based SVM. Given the +1/−1 binary response vector ***y***, the network-constrained SVM can be formulated as the addition of the hinge loss $\mathop {\sum}\nolimits_{i = 1}^n {{{\left[ {1 - {y_i}\left( {{\beta _0} + {\boldsymbol{x}}_i^{\rm{T}}\beta } \right)} \right]}_ + }}$ and the graph Laplacian constraint, where the subscript “+” denotes the positive part, i.e., *z*_+_ = max{*z*, 0}.

Semi-supervised learning methods can more conveniently explore the structures among both the genomic features and the patient samples by learning with the graph Laplacians,^39–41^ as shown in Fig. 3d. In the bipartite graph formulation introduced in the paper,^40^ gene expression data are represented as a bipartite graph with weighted edges between patient samples and genomic features. The bipartite graph captures the co-expression among the genes and the samples as bi-clusters in the graph such that both the sample clusters and feature modules are explored. In the hypergraph formulation introduced in the papers,^39, 41^ the gene expression data are represented as weighted hyperedges on the patient nodes, and a graph Laplacian on the hypergraph can be introduced for semi-supervised learning on the patient samples. An additional graph Laplacian of a protein–protein interaction (PPI) network is then introduced to incorporate network information among the genomic features.

It is also possible to regularize non-negative matrix factorization (NMF) models with a graph Laplacian,^42, 43^ as shown in Fig. 3e. NMF aims to find two non-negative matrices ***U***_*m* × *k*_ and ***H***_*n* × *k*_ whose product can accurately approximate the data matrix ***X*** with ***X*** ≈ ***UH*** ^*T*^. Combining the geometrically-based constraint with the original NMF leads to the graph-regularized NMF, where Tr(⋅) denotes the trace of a matrix.

### Preprocessing integration to detect network-based features

The preprocessing integration methods comprise two steps, as illustrated in Fig. 2b. First, the genomic profiles and the network are processed together to generate network-based features; second, standard learning models are applied with the network-based features for predictions. In this scenario, the integration of network and genomic data occurs before applying a learning model. The paper^44^ first proposed a graph algorithm to detect discriminative subnetworks for classification of patient samples. Highly discriminative genes are used as seed genes in a greedy search in a PPI network to find discriminative subnetworks, and then gene expression in each subnetwork is normalized as one feature value for classification with standard logistic regression. A similar approach was later proposed for application with features of discriminative pathways instead of subnetworks.^45^ In this approach, the gene expression in a pathway is normalized as one feature for the collection of pathways from a molecular signature database.^46^ The paper^47^ used disease-specific subnetworks as features, where a set of known disease genes are first mapped into the PPI network and then the subnetworks of the disease genes are identified as disease module features. The paper^48^ proposed implementing label propagation on the mutation data of each patient on a PPI network to generate network-smoothed features for classification of the patients. The paper^49^ proposed to find a small subnetwork to connect all differentially expressed genes in a PPI network and then use the genes in the subnetwork as features to classify patient samples. This setting is the Steiner tree problem in graph theory, and a heuristic algorithm coupled with randomization was designed to combine multiple suboptimal Steiner trees to find an optimum solution with a higher probability.

This category of algorithms is a very useful generalization of the earlier gene-set-based methods^50, 51^ since the network structures suggest dynamic modules among the genes rather than a fixed set. These modules can be data-specific and disease-specific for improved results. Thus, the data-driven subnetwork discovery introduced by these methods is a key improvement over previous studies.^50, 51^

### Post-analysis of oncogenic alterations in networks

The post-analysis integration methods also consist of two steps, as illustrated in Fig. 2c. First, the genomic profiles are analyzed to generate a list of oncogenic alterations; second, the detected alterations are analyzed in the network. In this post-analysis integration, the network information is integrated in the analysis after the oncogenic alterations are first detected by standard statistical methods. The purpose of these methods is to assess how cancer-driving alterations disrupt a normal cellular system by examining the influences on network components.

The circuit flow algorithm^52^ first identifies differentially expressed genes and then the genomic aberrations by mutations and copy number variations (CNVs) associated with the differential gene expression. Next, a current flow algorithm is applied to find causal paths from the causal genes (altered genes) to the target genes (differentially expressed genes) in a PPI network. Finally, the causal genes are selected by a set-covering algorithm to explain all the differentially expressed target genes.

HotNet^53^ first maps gene alterations in a gene network and then employs a diffusion kernel^54^ to build an influence graph with the edges weighted by the influence between each pair of genes. Then, a combinatorial problem is formulated to find the subnetworks of genes altered in a significant number of patients. Similarly, TieDIE^55^ and HotNet2,^56^ an extension of HotNet, apply network diffusion to analyze multiple types of genomic alterations, and NetPathID^57^ applies network diffusion to analyze CNVs in 16 types of cancers.

PARADIGM^58^ is a probabilistic graphical model framework used to model the gene transcription, translation and post-translational events. Each gene is modeled by a factor graph of DNA copy numbers, gene expression, protein levels and protein activities. The factor graphs of genes are connected based on their regulatory relations in a pathway. The genomic and proteomic data are analyzed in the graphical models for the inference of pathway activities in each patient to derive integrated pathway activity (IPA) scores. The significantly altered genes/pathways can be identified using the IPA scores.

The mutual exclusivity module (MEMo) method^59^ is another widely used method in the TCGA project. MEMo first builds a matrix representation of genes that are significantly altered by mutations or CNVs. Then, the altered genes are connected by their proximal in the HPRD PPI network.^7^ Finally, the cliques (a subgraph with all the gene pairs connected) are identified to analyze the mutual exclusivity in the patient data.

Signaling pathway impact analysis (SPIA)^60^ and mixed integer programming (MILP)^61^ are two examples of earlier pathway-based methods for genomic data analysis. SPIA applies an iterative algorithm similar to a random walk to measure the pathway perturbations in the regulatory network such that the impact of differentially expressed genes on a pathway can be evaluated.^60^ MILP is an optimization model to predict flux activity states of genes based on gene expression and a metabolic network.^61^

### Comparison of the methods

Network-based analysis of genomic data is based on the assumptions that cancer-driven aberrations often target different genes in the same pathway or subnetwork in the molecular network and that such systematic behavior can be observed as a coordinated change of genes’ functions in pathways or network modules. Network-based analysis is an effective approach because it has been observed that mutated genes in a cancer pathway can either co-occur in the same patients or be mutually exclusive among the patients, and the systematic behavior is a more detectable and interpretable signal for the assessment of functional impacts of the aberrations.^59^ It has also been shown that feature selection smoothed by graph Laplacian regularization based on the gene co-expression network is highly robust and generates more reproducible feature selections across independent datasets.^62^ Thus, the network-based approach is both well motivated and validated.

The three categories of methods have different relative advantages and disadvantages. Model-based integration methods are a fully supervised approach for both outcome prediction and subnetwork detection. The subnetworks are jointly discovered to contrast the control/case groups in the study based on a global optimization strategy, and thus these methods typically perform better in outcome prediction. In addition, the models can be tuned by a few clearly defined parameters, making it possible to train the models with cross-validation in contrast to the two-step methods in the other categories. The disadvantage is the need for more sophisticated optimization techniques, which are often less scalable. The preprocessing integration methods are more flexible in detecting customizable subnetwork features such that the detected features clearly reflect the hypothesized network-based characteristics. For example, the size and density of discriminative subnetworks can be precisely specified. However, it is not possible to guarantee that the detected subnetwork features are optimal features for prediction with the standard learning model in the second step. The post-analysis integration methods focus on associating mutations or other DNA aberrations with differential expression or certain other molecular phenotypes in the network context. Thus, these methods are highly informative regarding cancer mechanisms in the network.

In model-based integration, Graph LASSO is another choice of graph-based regularization other than the graph Laplacian regularizer.^63^ Graph LASSO imposes a LASSO loss on each pair of connected variables in the network rather than a squared error as with the graph Laplacian regularizer. The LASSO loss terms force the coefficients of the connected pairs to be identical such that the inconsistent pairs are “sparse.” In practice, the assumption can be too strong in networks with overlapping clusters. In addition, optimization of Graph LASSO-constrained models is generally challenging, while the graph Laplacian regularizer is a quadratic constraint that is relatively straightforward to optimize. Thus, Graph LASSO is a less common choice for network-based integration methods.

## Network-based methods for drug repositioning

**Fig. 4:**
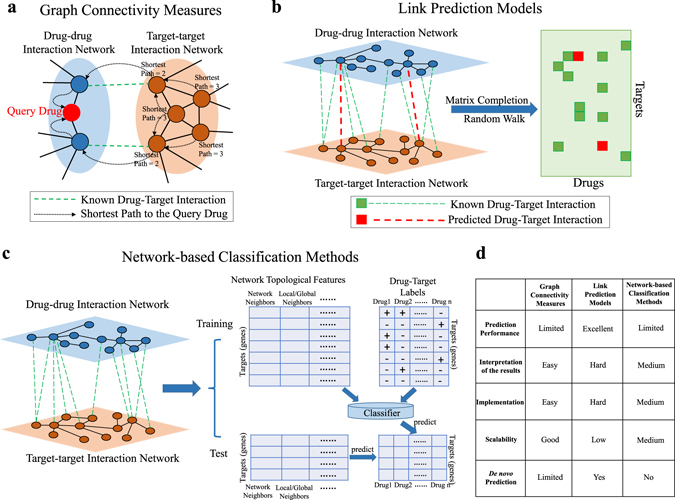
Methods for network-based drug repositioning. **a** Graph connectivity measures consider the local structures of the networks to predict drug–target interactions. This example shows the shortest path from each target node to the query drug (*red node*) in the graph. **b** Link prediction models predict the relations between drugs and targets based on the global structures of the known interactions in the networks with matrix completion or random-walk approaches. The known and predicted drug–target interactions are *green* and *red*, respectively, in the drug–target relation matrix. **c** Network-based classification methods first extract the network topological features for all the targets in the networks. For each drug, a classifier can be trained with the known targets of the drug as positive samples and the others as negative samples. The learned classifiers can then be used to predict the new targets in the test set for each drug. **d** The advantages and disadvantages of the methods in each category are compared

Network-based algorithms have also been developed for drug repurposing by exploring drug–drug similarities, drug–target relations and gene-gene relations. These methods can be largely classified into three categories, i.e., graph connectivity measures, link prediction models and network-based classification methods, as illustrated in Fig. 4. The methods reviewed under each category are also listed in Fig. 1b. Below, we describe and compare the methods in the three categories.

### Graph connectivity measures

The methods in this category are based on measuring the connectivity among the nodes in the graph, such as neighboring relations, the number of shared neighbors and shortest paths, to derive drug–drug, drug–target or drug–disease relations, as illustrated in Fig. 4a. Several early studies^64, 65^ showed that drugs sharing similar chemical structures, transcriptional responses following treatment and text mining analysis often share the same target, where the implication is that the drug–drug network based on the similarities can be used to reposition a drug for the targets of similar drugs. The paper^64^ derived drug–drug similarities based on mining the side-effect description from medical symptoms in the Unified Medical Language System ontology. The paper^65^ developed a method to predict similarities in terms of drug effect by comparing gene expression profiles following drug treatment across multiple cell lines and dosages. Both studies validated the correlation between drug–drug similarity and the likelihood of two drugs sharing a common protein target. Based on the observations, the paper^66^ proposed a recommendation technique for predicting drug–target relations based on the drug–drug similarity matrix ***W*** computed based on the structural similarity of the drugs and sequence similarity of their targets and the known drug–target matrix ***A***. By a simple multiplication (***R*** = ***WA***), the scores in matrix ***R*** can be used to derive a ranking of the candidate targets against each drug.

The paper^23^ performed a large-scale analysis of ~7000 genomic expression profiles in the Gene Expression Omnibus with human disease and drug annotations to create a disease–drug network consisting of drug–drug, drug–disease and disease–disease relations. The study shows that the derived disease–disease relations are highly consistent with the definition in the Medical Subject Headings disease classification tree and that the drug–disease relations can be used to generate hypothesized drug repositioning and side effects. The paper^6^ further generalized the inference to drug–disease proximity in the network by the hypothesis that an effective drug for a disease must target proteins within or in the immediate vicinity of the corresponding disease module in the molecular interaction network. They applied a shortest-path-based measure coupled with a randomization normalization technique to derive the drug–disease proximity scores for the inference.

A recent work in the paper^67^ performed a correlation analysis of disease modules and drug targets in the functional linkage network. The differentially expressed disease genes and the drug–target genes are first overlapped in the functional linkage network, and a mutual predictability score is then computed based on the neighboring relations among the genes to evaluate the repositioning of the drug for the disease.

### Link prediction models

Link prediction models predict the relations between drugs and targets based on the global structures of the known interactions in the networks with matrix completion or random-walk approaches, as illustrated in Fig. 4b. The paper^68^ predicted drug–target relations for drug repositioning based on a network of three types of relations: drug–drug structural similarity, target–target sequence similarity and drug–target relations from DrugBank.^26^ It was shown that exploring the network topology outperforms simple inference rules by graph connectivity measures such as similar drugs sharing the same target or similar targets sharing the same drug. The paper^69^ applied an information-flow approach on a heterogeneous network of drug–drug, disease–disease and target–target similarities along with the known disease–drug and drug–target relations. The algorithm iteratively updates the disease–drug and drug–target relations and converges to stationary scores for the prediction of their relations.

The paper^70^ introduced a bipartite graph-learning method based on kernel regression to learn a co-mapping of drugs in chemical space and targets (proteins) in genomic space into a common pharmacological space. In the pharmacological space, the correlation between compound-protein pairs can be conveniently calculated to predict their interactions for drug repositioning.

The paper^71^ proposed a collaborative matrix factorization method to factorize known drug–target relations to predict new relations constrained by the drug–drug similarity network and the target–target similarity network. The paper^72^ proposed a manifold regularization semi-supervised learning method in which two classifiers in drug space and target space are learned and then combined to give a final score for drug–target interaction prediction. The paper^73^ applied several random-walk methods on a heterogeneous network of drug–drug similarities, target–target similarities and drug–target relations such that the global structure among all the networks can be used to improve the prediction of new drug–target pairs.

### Network-based classification methods

Network-based drug repositioning can also be reformulated as a classification problem such that standard classification methods can be applied to predict the new targets of each drug, as illustrated in Fig. 4c. These methods first extract the network topological features for all the targets in the networks. For each drug, a classifier can be trained with the known targets of the drug as positive samples and the others as negative samples. The learned classifiers can then be used to predict the new targets in the test set for each drug. The paper^74^ proposed mapping disease-specific differentially expressed genes into a PPI network and using network topological features to detect new drug targets based on the known targets from the drug–target database by logistic regression. The paper^75^ also applied a supervised bipartite model to predict the probability of each drug–target interaction based on the known drug targets as labels and the target–target interactions as features, where the bipartite model was augmented with additional training samples from the neighboring drug–target relations.

The paper^76^ constructed a drug–drug kernel matrix based on chemical structure similarities and a target–target kernel matrix based on sequence similarities. For each drug, using the known targets as the positive training samples, an SVM classifier is built with the target–target kernel matrix to classify the candidate genes for new targets. In addition, for each target and using the known drugs as the positive training samples, an SVM classifier is built with the drug–drug kernel matrix to classify the drugs for new repositioned drugs. The paper^77^ adopted a similar approach with two additional advanced kernel methods, applying diffusion-types of kernels to integrate both the drug–drug kernel matrix and the target–target kernel matrix to predict the new targets of a drug or the new repositioned drugs for a target.

### Comparison of the methods

The three categories of methods have different relative advantages and disadvantages, as shown in Fig. 4d. Graph connectivity measures are straightforward to implement based on standard graph algorithms, and the prediction results are easy to interpret with the edges and the paths in the graph. However, the prediction performance is typically worse since only relatively local information of the networks is considered by the graph algorithms. Link prediction models retrieve the global structures of the networks to predict drug–target interactions for better prediction performance. The disadvantages are the lack of a satisfactory interpretation of the predictions and that the implementation of the models often relies on advanced optimization algorithms. When sophisticated optimization is required, the scalability can be poor. Network-based classification methods are more accurate for repositioning drugs with many known targets as the training samples but are not applicable to drugs with few or no known targets. The prediction results can be interpreted by the network topological features extracted from the networks, depending on the feature extraction strategy.

Another important aspect of the comparison is whether a method can generate de novo predictions for drugs with no known targets or gene targets with no known drugs. Graph connectivity measures are often more biased towards highly connected nodes in the graph such that new drugs or less-studied genes typically receive low rankings. Thus, de novo predictions are rarely made by graph connectivity measures. With no positive training pairs available, the network-based classification methods simply abandon the de novo cases. Link prediction models are often the most capable of making de novo predictions because global topological structures are generally less biased after proper normalization and control by randomization.

## Network-based analysis of TCGA mutation data and a case study on ovarian cancer

**Fig. 5:**
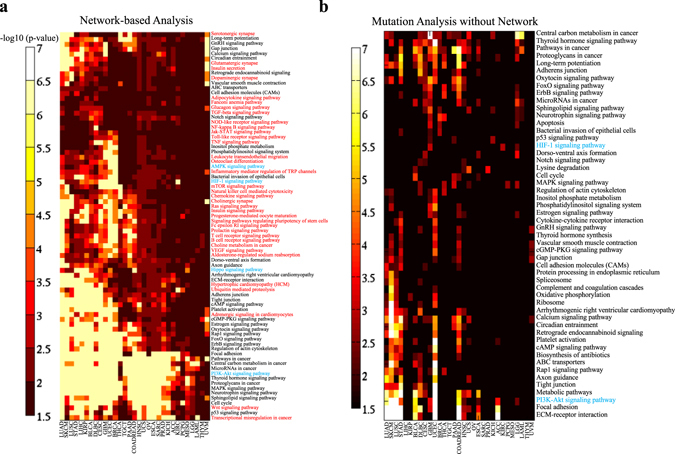
Network-based analysis of highly mutated pathways of 31 cancer types in TCGA data. The highly mutated pathways detected by **a** network-based analysis and **b** standard enrichment analysis. The pathways of interest in the discussion are highlighted in *blue*, and the pathways only enriched by network-based analysis are highlighted in *red*

To better discuss the network-based methods, we performed a network-based analysis of the mutated genes in the 31 cancer genome projects in TCGA^78–101^ and summarized the enriched KEGG pathways^102^ in Fig. 5. For the analysis, the mutation frequencies among the patients in the 31 TCGA provisional studies were downloaded from cBioPortal for Cancer Genomics.^103^ In the network-based analysis, label propagation (*λ* = 0.5)^48, 62^ as described in Table S2 in the Supplementary Information was applied to the HPRD PPI network^7^ in each cancer study to capture the highly mutated subnetworks. The initialization was the gene mutation frequency among the patients in each cancer study for label propagation. The summation of the stationary scores of the genes in a KEGG pathway is compared with the scores of 10,000 random gene sets of the same size to derive *p*-values. In the analysis without the network, the highly mutated genes in each cancer type are overlapped with KEGG pathways with enrichment analysis to derive *p*-values by hypergeometric test. This network-based analysis clearly detects more significantly mutated pathways than the analysis without using the network, as shown in Fig. 5a, b, respectively.

Interestingly, the network-based analysis in Fig. 5a indicates that the AMPK signaling pathway is affected in breast cancer (BRCA) and uterine corpus endometrial cancer (UCEC). Prior studies demonstrated that BRCA patients receiving metformin, a pharmacological activator of AMPK, showed complete pathologic response, implicating the role of AMPK in BRCA.^104^ Similarly, the loss of the AMPK activator LKB1 promotes endometrial cancer progression and metastasis,^105, 106^ implicating the AMPK pathway in endometrial cancer, and metformin inhibits endometrial cancer cell proliferation.^107^ The HIF-1 pathway has been predicted to be affected in renal clear cell carcinoma (KIRC), BRCA, endometrial cancer (UCEC), glioblastoma multiforme (GBM), cervical cancer (CESC), and lung cancer (LUAD), and these results are consistent with prior studies implicating the VHL/HIF-1 pathway in these cancers.^90, 108^ The Hippo pathway has been predicted to be affected in colorectal cancer, renal papillary carcinomas, stomach cancer, and liver cancer, and these results are consistent with recent cancer genomic studies.^97, 109^ Finally, the PI3K-Akt pathway has been identified as one of the most frequently affected pathways in several cancer types, and several components of this pathway were reported to be mutated or amplified in various cancer types.^110^ Collectively, these results suggest that network analysis can identify clinically relevant pathways that are altered in different cancer types.

**Fig. 6:**
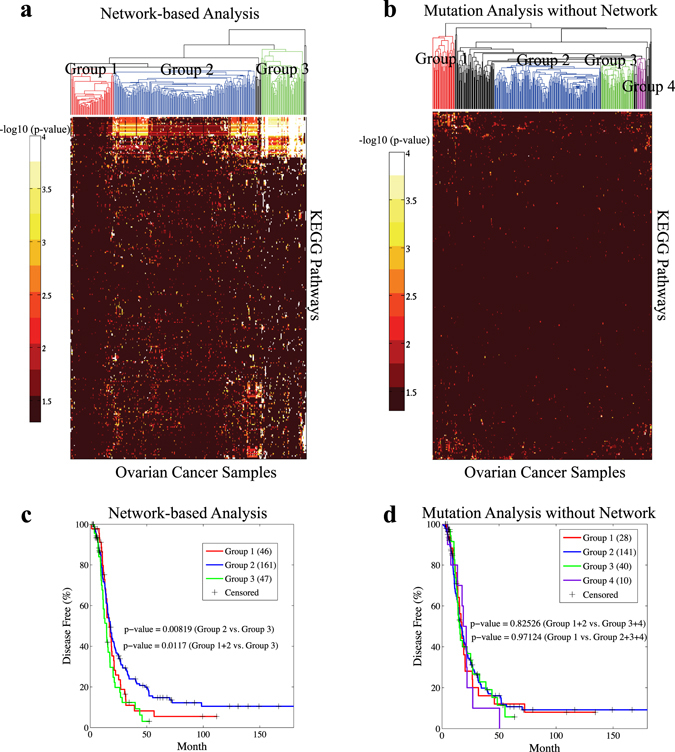
Network-based analysis of patient mutation data in TCGA ovarian cancer. The significantly mutated pathways in each patient detected by **a** network analysis and **b** the analysis of the original mutation data without the network. **c** The survival plot of the three groups detected by the network-based pathway analysis of the TCGA ovarian cancer patients. Derived by standard log-rank test, the *p*-values for comparing group 2 vs. group 3 and group 1 + group 2 vs. group 3 are both significant. **d** The survival plot of the groups detected by the analysis of the original mutation data of the TCGA ovarian cancer patients

In the case study on the ovarian cancer patients shown in Fig. 6, the mutation data of the 316 TCGA ovarian cancer patients were downloaded from the Xena Public Data Hubs.^111^ Similar to the study in the paper,^48^ label propagation (*λ* = 0.1) was applied on the same HPRD PPI network in each patient to detect the patient-specific highly mutated subnetworks. The initialization was 1 for the mutated genes and 0 for the other genes and then normalized to sum to 1. Similarly, the summation of the stationary scores of the genes in a KEGG pathway was compared with the scores of 10,000 random gene sets of the same size to derive the *p*-value. In the analysis without the network, the mutated genes in each patient are overlapped with KEGG pathways with enrichment analysis to derive *p*-values by hypergeometric test. Hierarchical clustering was applied to cluster the patients into three groups using the –log_10_ (*p*-values) as features. The network-based analysis informs a clustering of the patients by a significant relevance to survival (Fig. 6c). Notably, three subgroups of tumor samples can be identified from the network-based analysis shown in Fig. 6c, compared to four subgroups in the mutation-based analysis without the network in Fig. 6d. Although subgroups identified by mutation-based analysis without the network show no significant association with disease-free survival, two of the subgroups detected by the network-based analysis (Subgroup 1 and Subgroup 3) show significant association with disease-free survival relative to Subgroup 2. Interestingly, Subgroup 1 has the highest copy number alterations, whereas Subgroup 3 has the highest number of pathway alterations. These results are analogous to the spectrum of somatic alterations described by ref. 112. Although those authors placed ovarian cancer in class C, defined by extensive copy number alterations, the spectrum of somatic alterations can be further described as subgroups with higher copy number changes, mixed, and higher mutations within ovarian cancer. This case study shows that via network analysis, several subtypes of ovarian cancer can be grouped together for further assessment of clinical values, such as occurrence, relapse and treatment resistance. This information may also be valuable for the design or assessment of treatment strategies. Collectively, the network analysis unveils important cancer pathways and their correlation to subtypes of cancers that would not be identifiable by original mutation data analysis.

## Discussion

Precision oncology tailors cancer treatment and repositions drugs based on personal genomic information. There are several promising aspects of the application of network-based analysis in precision oncology. With a network to capture the molecular organization in the cellular system, genomic data analysis is both more accurate and descriptive. The smoothness constraint introduced into the model-based integration methods is helpful in eliminating false positives and false negatives in high-dimensional genomic data. The network analysis identifies molecular targets in the context of pathways or interaction partners in a subnetwork that are interpretable for molecular mechanisms. For example, in the case study in Fig. 6a, the mutation information of each individual patient is propagated on the PPI network to detect the patient-specific subnetwork and improve the quality of the patient clustering by a significant relevance to survival. As a consequence, network-based analysis often reports consistent marker genes across different studies of the same cancer^40^ or more comparable results in pan-cancer analysis.^56^ Collectively, it is evident that network-based methods employ molecular and biomedical networks to extract useful personal genomic information, and build better predictive models for target identification, phenotype prediction and drug repositioning.

Conceptually, network-based analysis also adopts mutation patterns that are mutually exclusive or co-occurring. Mutually exclusively mutated genes are often located on the same pathway, and network analysis propagates the mutually exclusive signals to identify the pathway by a significant signal. Co-occurring mutated genes in a pathway/dense network module also mutually strengthen the mutation signals. The results in Fig. 6 clearly support that the mutation patterns are accurately captured in the case study on ovarian cancer by label propagation.

In drug repositioning, both molecular networks and drug–drug or phenotype similarity networks play important roles. It has been repeatedly observed that genes associated with the same (or similar) diseases tend to lie in a dense module in the PPI network. This observation has motivated effective network-based methods to predict new disease genes.^43^ The analysis of gene modules in the PPI of similar diseases has also suggested associations between diseases and gene functions or pathways.^43^ When drug targets and disease genes are analyzed together in the PPI network, their proximities are useful for drug repositioning.^6^

The methods compared in Figs. 2 and 4 have different relative advantages and disadvantages. The considerations involve a variety of key properties, including the performance of the methods, the interpretation of the results, the difficulty of implementation, the scalability to genome-wide analysis, and the characteristics of the training data. The appropriate choice of a network-based method for a particular analysis can be customized based on the information gained from these comparisons. For example, drugs with more known targets can be repositioned by the network-based classification models, while drugs with no known targets in the candidates can be repositioned by the link prediction methods. Depending on whether the analysis must be highly scalable to a huge network, simple graph connectivity measures or link prediction methods can be used.

In the application of network-based analysis, there are also several practical issues and limitations.

1. Molecular networks often contain biased information. Well studied genes tend to have more connections in the PPI network, and they are also targets of more drugs and are associated with more disease phenotypes. Typically, it is important to exercise normalizations and repeat the experiments on randomized networks to assess the statistical significance of the results. The biases also prevent the prediction of de novo disease genes or target genes if the gene has no association with known diseases or is not a target of any drug.^25^
2. The empirical results of network-based methods rely on tuning parameters. The parameters often balance how much belief is imposed on the network topologies. When excessive weights are assigned to the network topology, there will be an “over-smoothing” effect such that nearly uniform scores are expected among the genes in even large and sparse neighborhoods. Thus, a proper procedure for determining the appropriate (optimal) parameters is critical, for example, by applying cross-validation and wet-lab validation.^36^
3. Commonly, a molecular network describes a general relation, such as protein–protein physical interaction or functional linkage. In some cases, the relations can be either positive or negative, e.g., gene co-expression. A practical approach is to apply a signed graph Laplacian.^113^ The models applied with a signed graph Laplacian can be solved in a manner similar to those with the normal graph Laplacian by the same algorithms.

Finally, this article targets the scope of precision oncology, including steps for understanding cancer mechanisms, finding targets and repositioning drugs, while previous survey studies have focused on detecting cancer-driven aberrations and understanding of the aberrations in molecular networks/pathways.^4, 114, 115^ This article also surveys several categories of algorithms, including model-based integration and preprocessing integration with machine learning methods, while previous reviews^4, 114, 115^ primarily surveyed the methods in one of the three categories, namely, post-analysis integration of oncogenic alterations in networks. Thus, this article offers a different scope and a more comprehensive survey of computational methods.

## Future directions

Several challenges remain in the application of network-based analysis in precision oncology. These challenges concern the data quality, deployment for research or clinical use, and scalability of network analysis.

To precisely model the molecular interactions and drug–target relations, networks of better quality are required. It is known that most molecular networks and drug–target databases are incomplete and biased towards well-studied proteins/genes. Thus, continuing effort on the improvement of the networks with additional experimental data is important. In addition, network modeling with higher resolution is also crucial to model complex molecular functions at higher precisions. For example, proteins are present in the isoforms of genes, and thus isoform–isoform interactions are the true interactions to model in a network^116–118^; mutations or other structure variations of a protein can also change the protein–protein binding or drug–protein docking in a specific tumor. Furthermore, even within each tumor, heterogeneous cell populations exist, and the drug targets and molecular interactions could be different for each cell population if measured by single-cell RNA sequencing.^119^ To partially address this issue, several computational methods for quality control of PPI screening have been proposed to reduce the number of false-positive and false-negative PPIs due to spurious errors and systematic biases from the high-throughput techniques.^120, 121^ Currently, it is still impossible to construct these more accurate networks at a large scale due to the limitation of the current high-throughput experimental methods for measurement of molecular interactions or drug screening.

While many network-based methods have been developed to support precision oncology, the implementations of the methods are independent, with non-standardized tools that are never easily accessible as a useful collection to oncologists for research or clinical use. Thus, there is a strong need to develop a software platform that integrates standardized biomedical, biological network data, and analytic software components to support comprehensive network-based analysis of patient genomic data and drug repositioning for precision oncology. This platform should be based on a sophisticated system design to meet oncologists’ requirements and support customization of the analysis pipeline. The concept of part of such a platform was proposed in the paper^5^ as an integrative network-based infrastructure to identify new druggable targets and repositionable drugs through the targeting of significantly mutated genes identified in human cancer genomes. In the future, the existing tools can be reimplemented as apps on a platform such as Cytoscape^122^ or another software environment similar to GALAXY for NGS data analysis^123^ to facilitate the development and deployment of the software system for precision oncology.

Finally, scalability is always an issue in network-based analysis since it is common to model millions of genomic features, hundreds of thousands of drugs and tens of thousands of phenotypes in a very large network. For example, in an isoform–isoform interaction network, hundreds of thousands of nodes are contained in a single graph that cannot be loaded onto a computer with less than 100 GB of memory. Such big-data analysis will require more scalable algorithms and efficient computing platforms. For example, the standard label propagation can be applied to low-rank approximations of big graphs, enabling work with networks of millions of nodes.^124, 125^ Parallel implementations of the network-analysis methods, especially the optimization algorithms in those model-based approaches, are also necessary.

## Electronic supplementary material

Supplementary

## Electronic supplementary material

**Supplementary Information** accompanies the paper on the *npj Precision Oncology* website (doi:10.1038/s41698-017-0029-7).
