# Supplementary material for: Network-based machine learning and graph theory algorithms for precision oncology
Source: NPJ Precis Oncol. 2017 Aug 8;1:25. doi: 10.1038/s41698-017-0029-7 (PMC5871915; doi:10.1038/s41698-017-0029-7)
Supplement: Supplementary file 1 — Supplementary [file 41698_2017_29_MOESM1_ESM.pdf]

Network-based Machine Learning and Graph Theory Algorithms for Precision Oncology

Wei Zhang[1], Jeremy Chien[2], Jeongsik Yong[3] and Rui Kuang[1]

[1]*Department of Computer Science and Engineering, University of Minnesota Twin Cities, Minneapolis, Minnesota, United States of America*

[2]*Department of Cancer Biology, University of Kansas Medical Center, Kansas City, Kansas, United States of America*

[3]*Department of Biochemistry, Molecular Biology and Biophysics, University of Minnesota Twin Cities, Minneapolis, Minnesota, United States of America*

Table S1: Glossary

Terms	Description
**directed graph**	all the edges are directed from one vertex to another in a directed graph
**bipartite graph**	edges in a bipartite graph only connect nodes in two disjoint sets
**hypergraph**	a graph which edges are sets of any number of vertices
**directed acyclic graph**	a directed graph with no directed cycle
**factor graph**	a bipartite graph representing the factorization of a function with two types of nodes (variables and factors)
**regularization**	introduction of an additional term to an objective/loss function of a statistical model for better generalization to new data
**spectral graph theory**	the study of the characteristics of the adjacency matrix or the Laplacian matrix associated with the graph
**supervised learning/approach**	a type of machine learning algorithm that build a model from labeled training data to make predictions on the unlabeled test data
**semi-supervised learning**	make use of both labeled and unlabeled data for training a machine learning model
**LASSO**	Least Absolute Shrinkage and Selection Operator; a regularization technique that performs sparse variable selection
**elastic-net**	a regularization technique that linearly combines the LASSO and ridge penalties
**logistic regression**	a linear classification model using logistic output
**Support Vector Machine (SVM)**	a large-margin based classifier that finds an optimal hyperplane to separate two classes
**bi-clustering**	a data mining technique which simultaneously clustering the rows and columns of a matrix
**label propagation**	a semi-supervised learning algorithm for label inference based on a graph structure
**Steiner tree problem**	find the minimum weight tree spanning through all the vertices in given subset in a graph
**heuristic algorithm**	a technique designed to find an approximate solution close to the optimal one more quickly than the methods finding the optimal solution
**random walk**	a stochastic process describing a path of a succession of random steps on a graph
**cross-validation**	estimate the performance of a predictive model by testing on a holdout labeled data set in addition to training and test data
**matrix completion**	the task of filling the missing entries in a matrix based on some error measures
**kernel function**	a positive semi-definite function to compute the pairwise similarity between two feature vectors
**diffusion kernel**	a special class of exponential kernels on graphs
**network diffusion**	calculate an overall network proximity by simulating the diffusion of a value throughout a network
**kernel regression**	a regression method based on kernel functions to allow non-linear relation between the random variables
**hierarchical clustering**	a clustering method to build a hierarchy of clusters of the samples

Table S2: Network-based Machine Learning Models.

**Base Model**	**Objective function**	**Definitions**
Linear regression [34]	$\mathcal{L}(\boldsymbol{\beta}\|\lambda_1,\lambda_2) = \|\|\boldsymbol{y} - \boldsymbol{X}^T\boldsymbol{\beta}\|\|^2 + \lambda_1\|\boldsymbol{\beta}\|_1 + \lambda_2\boldsymbol{\beta}^T\boldsymbol{L}\boldsymbol{\beta}$	
Cox regression [36]	$\mathcal{L}(\boldsymbol{\beta}, h_0\|,\lambda_1,\lambda_2) = \sum_{i=1}^{n}\left\{-\exp(\boldsymbol{x}_i^T\boldsymbol{\beta})H_0(t_i) + \delta_i\left[\log(h_0(t_i)) + \boldsymbol{x}_i^T\boldsymbol{\beta}\right]\right\}$ $-(\lambda_1\boldsymbol{\beta}^T\boldsymbol{\beta} + \lambda_2\boldsymbol{\beta}^T\boldsymbol{L}\boldsymbol{\beta})$	t_i: observed or censored survival time for the ith patient. $h_0(t)$: baseline hazard function. $H_0(t_i) = \sum_{t_k \le t_i} h_0(t_k)$. δ_i: indicator of the survival time t_i is observed or censored.
Logistic regression [37]	$\mathcal{L}(\boldsymbol{\beta},\beta_0\|\lambda_1,\lambda_2) = \sum_{i=1}^{n}\left\{y_i\log p(\boldsymbol{x}_i) + (1-y_i)\log\left(1-p(\boldsymbol{x}_i)\right)\right\}$ $-(\lambda_1\|\boldsymbol{\beta}\|_1 + \lambda_2\boldsymbol{\beta}^T\boldsymbol{L}\boldsymbol{\beta})$	$\boldsymbol{y} = (y_1,...,y_n)^T$ with $y_i \in \{1,0\}$. β_0: intercept. $p(\boldsymbol{x}_i)$: the probability that the ith sample is in class 1.
Support vector machine [38]	$\mathcal{L}(\boldsymbol{\beta},\beta_0\|\lambda_1,\lambda_2) = \sum_{i=1}^{n}[1 - y_i(\beta_0 + \boldsymbol{x}_i^T\boldsymbol{\beta})]_+ + \lambda_1\boldsymbol{\beta}^T\boldsymbol{\beta} + \lambda_2\boldsymbol{\beta}^T\boldsymbol{L}\boldsymbol{\beta}$	"+": the positive part, i.e., $z_+ = \max\{z,0\}$. $\boldsymbol{y} = (y_1,...,y_n)^T$ with $y_i \in \{1,0\}$.
Bipartite-graph-based learning [40]	$\mathcal{L}(\boldsymbol{f},\boldsymbol{\beta}\|\lambda) = \|\|\boldsymbol{f}\|\|^2 + \|\|\boldsymbol{\beta}\|\|^2 + 2f^T\boldsymbol{S}\boldsymbol{\beta} + \lambda\|\|\boldsymbol{f} - \boldsymbol{f}^{(0)}\|\|^2$	$\boldsymbol{X}^+$: non-negative adjacency matrix of the bipartite graph representation of $\boldsymbol{X}$ (40). Bipartite graph: $\boldsymbol{S} = \boldsymbol{D}_{\boldsymbol{c}}^{-\frac{1}{2}}\boldsymbol{X}^+\boldsymbol{D}_{\boldsymbol{r}}^{-\frac{1}{2}}$, where $\boldsymbol{c}$ and $\boldsymbol{r}$ are column and row sum of $\boldsymbol{X}^+$.
Hypergraph-based learning [39,41]	$\mathcal{L}(\boldsymbol{f},\boldsymbol{\beta}\|\lambda_1,\lambda_2) = \boldsymbol{f}^T(\boldsymbol{I} - \boldsymbol{D}_{\boldsymbol{v}}^{-\frac{1}{2}}\boldsymbol{H}\boldsymbol{D}_{\beta}\boldsymbol{D}_{\boldsymbol{e}}^{-1}\boldsymbol{H}^T\boldsymbol{D}_{\boldsymbol{v}}^{-\frac{1}{2}})\boldsymbol{f}$ $+\lambda_1\|\|\boldsymbol{f} - \boldsymbol{f}^{(0)}\|\|^2 + \lambda_2\boldsymbol{\beta}^T\boldsymbol{L}\boldsymbol{\beta}$	$\boldsymbol{H}$: hyper-graph adjacency matrix constructed from $\boldsymbol{X}$ ([39,41]). $\boldsymbol{v}$ and $\boldsymbol{e}$ are column (vertex) sum and row (hyperedge) sum of $\boldsymbol{H}$.
NMF [42,43]	$\mathcal{L}(\boldsymbol{U},\boldsymbol{H}\|\lambda) = \|\|\boldsymbol{X} - \boldsymbol{U}\boldsymbol{H}^T\|\|^2 + \lambda\mathrm{Tr}(\boldsymbol{U}^T\boldsymbol{L}\boldsymbol{U})$	Nonnegative matrices $\boldsymbol{U} = [u_{ik}] \in \mathbb{R}^{m\times k}$ and $\boldsymbol{H} = [h_{jk}] \in \mathbb{R}^{n\times k}$.
Label Propagation (LP) [62]	$\mathcal{L}(\boldsymbol{\beta}\|\lambda) = \|\|\boldsymbol{\beta} - \boldsymbol{\beta}^0\|\|^2 + \lambda\boldsymbol{\beta}^T\boldsymbol{L}\boldsymbol{\beta}$	$\boldsymbol{\beta}^0$: initial coefficients.
